# Decreased Serum NCAM Levels Associated with Cognitive Impairment in Vascular Dementia

**DOI:** 10.1155/2021/2792884

**Published:** 2021-08-31

**Authors:** Jun Zhao, Wei Lu, Junshan Li, Lei Liu, Xiumin Zhao

**Affiliations:** ^1^Department of Neurology, Qingpu Branch of Zhongshan Hospital, Fudan University, Shanghai 201700, China; ^2^Department of Intensive Rehabilitation, Shandong Provincial Third Hospital, Cheeloo College of Medicine, Shandong University, Jinan, Shandong Province 250000, China; ^3^Department of Gastroenterology, Shandong Provincial Third Hospital, Cheeloo College of Medicine, Shandong University, Jinan, Shandong Province 250000, China; ^4^Department of Neurology, Shandong Provincial Third Hospital, Cheeloo College of Medicine, Shandong University, Jinan, Shandong Province 250000, China

## Abstract

**Objective:**

Neural cell adhesion molecule (NCAM), a glycoprotein widely distributed in the brain, has recently been shown to regulate neuroplasticity. However, the role of NCAM in vascular dementia (VaD) is still unclear. The purpose of this study is to determine whether NCAM is involved in the course of VaD.

**Methods:**

Continuous recruitment of VaD patients and control population to join this study. Doctors or nurses are responsible for collecting their clinical characteristics including age, gender, formal education, heart rate, supine systolic blood pressure, supine diastolic blood pressure, fasting glucose, high-density lipoprotein, and low-density lipoprotein. Each participant received the Montreal Cognitive Assessment (MoCA) scale after being enrolled in the group. At the same time, their peripheral blood was collected, and their serum NCAM levels were measured by enzyme-linked immunosorbent assay (ELISA).

**Results:**

98 VaD patients and 83 age- and sex-matched controls were enrolled. There was no significant statistical difference between the VaD group and the control group in terms of the comparison of clinical characteristics (*p* > 0.05). The MoCA score of VaD patients was significantly lower than that of the controls (27.9 ± 1.4 vs. 23.0 ± 2.1 points, *p* < 0.001). In addition, the circulating NCAM level of VaD patients was also significantly lower than that of controls (21.7 ± 3.8 vs. 17.6 ± 4.2 ng/mL, *p* < 0.001). The circulating NCAM level of VaD patients was significantly positively correlated with MoCA score (*r* = 0.285, *p* = 0.026). After adjusting for clinical characteristics, circulating NCAM levels are still an independent pathogenic factor of VaD (regression coefficient = 0.223, *p* = 0.034).

**Conclusions:**

VaD patients have low circulating NCAM levels, which can be used as a potential predictor of VaD.

## 1. Introduction

Vascular dementia (VaD) is a general term describing brain damage caused by cerebral blood flow disorders, which can cause problems in reasoning, judgment, learning, planning, memory, and other thinking processes [1–3]. About 15-20% of people with dementia will develop VaD. It is the second most common dementia among people over 65 [4]. As people's lifestyle changes and life pressure increases, the prevalence of VaD remains high. The latest epidemiological studies show that the number of people diagnosed with dementia will rapidly increase to 131 million by 2050 [5, 6]. Hypertension, diabetes, smoking, hyperlipidemia, and arrhythmia are common causes of VaD [7, 8]. However, current treatments based on controlling risk factors cannot fundamentally alleviate VaD.

Wilson discovered the phenomenon of cell adhesion in 1907, and the neural cell adhesion molecule (NCAM), a special cell adhesion molecule, was discovered by Jørgensen and Bock in 1974, and it was originally named to synaptic membrane protein D2 [9, 10]. NCAM is encoded by a gene located on chromosome 11. Due to alternative splicing, it is divided into three main subtypes according to its molecular weight: NCAM-120, NCAM-140, and NCAM-180 [11, 12]. The intracellular parts of these three NCAM subtypes are different, so they can show different expression patterns and functions [13]. NCAM, also known as CD56, belongs to the immunoglobulin superfamily and is a glycoprotein mainly expressed in neurons and glial cells [14]. NCAM plays an important role in neurodevelopment, neuromigration, synaptic growth, and neuroplasticity and affects the formation of learning and memory [15].

In the decades after the discovery of NCAM, its role in a variety of neuropsychological and neurodegenerative diseases has been widely reported. NCAM has also been isolated and identified, and its mimic peptide is also considered a neuroprotective agent. However, the relationship between NCAM and VaD, the second largest type of dementia, is still unclear. The purpose of this study is to explore the relationship between NCAM and the cognitive function of VaD patients.

## 2. Methods

### 2.1. Study Subjects

From January 2019 to December 2020, VaD patients treated in the outpatient and ward of Shandong Provincial Third Hospital were registered. The diagnosis of VaD patients is based on the Diagnostic and Statistical Manual of Mental Disorders V (DSM-5) and National Institute for Neurological Disorders and Stroke (NINDS-AIREN), made by experienced neurologists. All subjects in the group were examined by computed tomography (CT) or magnetic resonance imaging (MRI). The inclusion criteria are as follows: (1) age over 50 years old; (2) no intracranial hemorrhage, deformity, or tumor in imaging examination; and (3) meet the diagnostic criteria of VaD. The exclusion criteria are as follows: (1) other causes of dementia, such as Alzheimer's disease (AD) and Lewy body dementia (DLB); (2) acute phase of new stroke; (3) history of previous head trauma or surgery; (4) severe psychological or mental illness; (5) dependence on drugs, alcohol, or heroin; (6) taking nootropics such as donepezil, rivastigmine, and sodium oligomannate (GV971); (7) unable to complete neuropsychological tests; and (8) individuals or the family refused to sign the informed consent. In addition, healthy volunteers without cognitive impairment were recruited as controls. All participants or their family members were informed of this study and agreed to participate in the study. The protocol of this study complies with the Declaration of Helsinki and has been approved by the local ethics committee. All research methods are strictly implemented in accordance with the relevant guidelines.

### 2.2. Clinical Characteristics Collection

After the study subjects were enrolled in the group, specialized medical staff were responsible for collecting their clinical characteristics. The collected clinical characteristics include age, gender, formal education, heart rate, supine systolic blood pressure, supine diastolic blood pressure, fasting glucose, high-density lipoprotein, and low-density lipoprotein. The age, gender, and formal education information is provided by the participant or a familiar caregiver. The remaining biochemical indicators are tested in the hospital laboratory according to standardized biochemical methods. All clinical characteristics were collected within 48 hours after enrollment and carefully recorded for analysis.

### 2.3. Cognitive Function Evaluation

The Montreal Cognitive Assessment (MoCA) is widely used as a screening test for highly educated elderly people with mild cognitive impairment and is generally considered to be suitable for subjects who have been educated for more than 13 years. Due to the low average education level of the elderly in China, our study adopted the MoCA basic test (MoCA-B). The purpose of revising the MoCA test is to make it suitable for individuals with lower education levels. “MoCA-B” is a slightly modified version of language and culture after review and approval by the creator of MoCA, calculation, concentration, executive function, conceptual thinking, and visual perception [16]. Like MoCA, the total score of the MoCA-B scale is 30 points, and a score lower than 26 points is considered to have cognitive impairment [17]. MoCA-B is online for free use by clinicians Dr. Ziad Nasreddine. It includes nine cognitive dimensions: language, direction, attention, and memory (http://www.mocatest.org/).

### 2.4. Biomarker Assessment

The circulating levels of NCAM are detected by enzyme-linked immunosorbent assay (ELISA). Commercial ELISA detection reagents were purchased from MyBioSource ELISA kit (MyBiosource, Inc., San Diego, CA, USA). The detection of this reagent adopts the sandwich method, and its detection sensitivity is 1.0 ng/mL, and the detection range is 3.12 ng/mL-100 ng/mL. The peripheral blood of the participants was collected, left at room temperature for 20 minutes, and then centrifuged at 1000 g for about 20 minutes. Carefully collect the supernatant to obtain the serum, and test it immediately or store the sample at -80°C to avoid repeated freezing and thawing [18]. The experiment sets up standard wells, sample wells, and blank/control wells, and each sample is measured repeatedly 3 times. The detection method is based on the product specification.

### 2.5. Statistical Methods

The statistics of measurement data are expressed as mean ± standard deviation, and the statistics of count data are expressed as *n* (%). Two independent samples *t*-test or *X*2 test was used for the uniformity of baseline data in the two groups. The bivariate Pearson and Spearman correlation coefficient was used to assess the correlation between the MoCA score and clinical characteristics. Multivariate regression analysis was used to evaluate the causal relationship between serum circulating NCAM levels and VaD after adjusting for clinical characteristics. All analyses are done with SPSS software package (IBM SPSS Statistics for Windows, Version 20.0). All hypothesis tests are significant with a two-tailed *p* value < 0.05.

## 3. Results

### 3.1. Clinical Characteristics of All Participants

**Figure 1:**
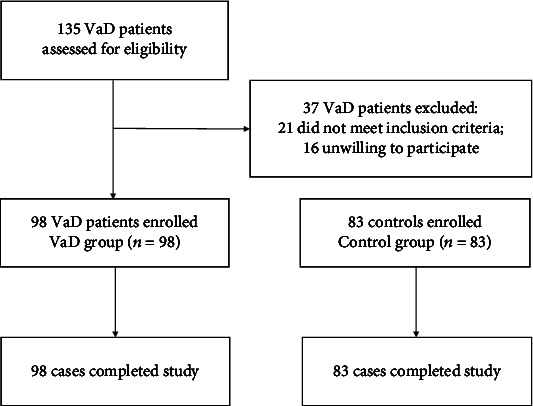
Flow diagram of the study. VaD: vascular dementia.

From January 2019 to December 2020, 135 VaD patients were screened in the outpatient and ward of Shandong Provincial Third Hospital. After screening, 37 people were excluded, of which 21 did not meet the inclusion criteria, and the remaining 16 refused to participate. This leaves 98 VaD participants who meet all the inclusion criteria, and they are assigned to the VaD group (*n* = 98). We enrolled 83 people without cognitive impairment as the control group (*n* = 83). In the end, all participants in the group completed the study and provided data for analysis. The flow diagram of the study is shown in Figure 1.

The clinical characteristics of the two groups are similar, and there is no significant difference between them (*p* > 0.05). These similar clinical characteristics include age, gender, formal education, heart rate, supine systolic blood pressure, supine diastolic blood pressure, fasting glucose, high-density lipoprotein, and low-density lipoprotein, as shown in Table 1.

**Figure 2:**
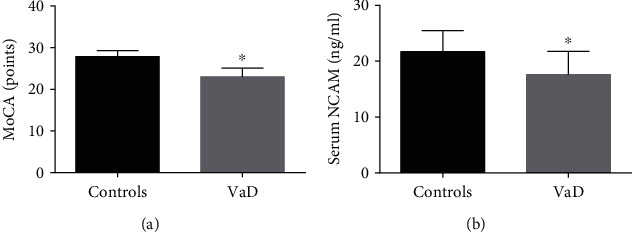
The MoCA scores and circulating levels of NCAM in all subjects: (a) MoCA scores; (b) circulating levels of NCAM. Compared with the controls, ^∗^*p* < 0.05.

However, the MoCA score of VaD patients was significantly lower than that of the control (27.9 ± 1.4 vs. 23.0 ± 2.1 points, *p* < 0.001). In addition, the circulating NCAM level of VaD patients was also significantly lower than that of controls (21.7 ± 3.8 vs. 17.6 ± 4.2 ng/mL, *p* < 0.001). The significant differences in MoCA scores and circulating serum NCAM levels between the two groups are shown in Figure 2.

### 3.2. Bivariate Correlation Analysis

To evaluate the correlation between clinical characteristics and cognitive function, we did a bivariate correlation analysis. As shown in Table 2, the bivariate analysis between age, gender, formal education, heart rate, supine systolic blood pressure, supine diastolic blood pressure, fasting glucose, high-density lipoprotein, low-density lipoprotein, and MoCA score shows that they are not significantly related. However, there was a significant positive correlation between circulating NCAM concentration and MoCA score (*r* = 0.258, *p* = 0.026).

### 3.3. Multivariate Regression Analysis

In order to further verify the correlation between clinical characteristics and cognitive function, we did a multivariate regression analysis to eliminate the influence of confounding factors on the conclusion as much as possible. After correction for age, gender, formal education, heart rate, supine systolic blood pressure, supine diastolic blood pressure, fasting glucose, high-density lipoprotein, and low-density lipoprotein, circulating NCAM levels can still significantly affect the cognitive function of VaD patients (regression coefficient = 0.223, *p* = 0.034). The results of the multivariate analysis are summarized in Table 3.

## 4. Discussion

In our study, we tested the differences in circulating NCAM levels and cognitive function between VaD patients and control groups. The results showed that the MoCA score and circulating NCAM levels in VaD patients were significantly reduced. These results suggest that low circulating NCAM levels may be related to the pathogenesis of VaD. In order to evaluate the possible link between VaD and circulating NCAM level, we did a correlation and regression analysis between circulating NCAM level and MoCA score. The results showed that even after adjusting for confounding factors, there was still a significant positive correlation between MoCA scores and circulating NCAM levels. As far as we know, there are currently limited papers on the correlation between circulating NCAM levels and VaD.

Cell adhesion molecule (CAM) is a ligand that participates in cell-cell recognition in vertebrates and can participate in pathophysiological processes such as the selective binding of axons, the selection of synaptic targets, or cell separation [19]. In the nervous system, many membrane glycoproteins or transmembrane proteins are identified as CAM proteins. NCAM is the first CAM protein identified as mainly expressed in the nervous system and can affect various cellular events in the nervous system during development or after maturation [20]. There are three main forms of NCAM. Among them, the large molecular weight is transmembrane proteins, while the small molecular weight NCAM-120 is attached to the membrane through glycosylphosphatidylinositol anchors [21]. NCAM can become soluble NCAM after being digested by restriction enzymes, which is widely present in the brain, cerebrospinal fluid (CSF), and plasma [22]. The mechanism of NCAM's biological effects in the body is complicated. On the one hand, NCAM mediates cell adhesion through homotypic and heterotypic interactions, thereby activating transmembrane signals and triggering calcium influx [23]. On the other hand, NCAM mediates the adhesion of cells to other cells or extracellular matrix and affects cell migration, neurite extension, and synapse formation by activating intracellular signaling pathways [24]. In addition, polysialic acid is thought to affect its biological functions in vivo and in vitro by regulating the cell adhesion activity of NCAM [25].

NCAM may play an important role in a series of neurological diseases. Gnanapavan's research team compared the differences in CSF NCAM levels in control populations with benign intracranial hypertension, multiple sclerosis, cases of AD, motor neuron disease, and meningitis and found that its levels in multiple sclerosis, AD, and meningitis are reduced [26]. The autopsy results of patients with refractory temporal lobe epilepsy showed that the expression of highly salivated NCAM in the hippocampus and entorhinal cortex increased, indicating that NCAM is involved in the remodeling of neuronal circuits in patients with epilepsy [27]. In the hippocampus of patients with schizophrenia, PSA-NCAM immunoreactivity is reduced, and there are more embryonic structures of NCAM, indicating that the plasticity of NCAM in the brain has changed, which further affects neurodevelopment [28]. Interestingly, NCAM is undetectable in normal muscle fibers, but it is abundantly expressed in denervated and regenerated muscle fibers, suggesting that NCAM can participate in the pathological process of neuromyopathy by promoting nerve regeneration [29, 30]. In addition, the relationship between CAM and stroke has long been discovered, but clinical trials for CAM targeted therapy have not been successful, indicating that the pathogenic mechanism of CAM has not been fully elucidated [31].

Accumulated evidence indicates that NCAM is a key regulator of synaptic plasticity, which can affect learning, memory, and cognitive function [32]. Indian scholars have found that the expression of neuroplasticity marker NCAM is increased in patients with bipolar disorder and is related to the severity of the disease [33]. German researchers confirmed that NCAM regulates synaptic plasticity and learning ability by inhibiting the signal transduction of NMDA receptors containing GluN2B [34]. A recent multinational joint study showed that NCAM plays an important neuroprotective role in the associative memory of C. elegans and humans, which proposes new therapeutic targets for memory-related diseases [35]. The effect of NCAM on neuroplasticity has been verified in cognitive impairment-related diseases or models. Scientists from Denmark found that an NCAM-derived peptide reduces the characteristic neuropathological changes and cognitive impairment induced by A*β*25-35 [36]. Clinical studies in Argentina have also shown that NCAM may be involved in the pathogenesis of AD disease and can be used as a differential diagnostic marker for the disease [37]. However, the role of NCAM in VaD has not been reported yet.

The advantage of our research is that we reported for the first time that NCAM is involved in the pathogenesis of VaD. However, our research also has some limitations. First of all, our study is a small cross-sectional study, lacking intervention studies for diagnostic targets. Secondly, VaD patients have different disease durations, which makes it impossible for us to track the dynamic changes of NCAM in the course of VaD disease. Finally, the etiology of VaD is different, which may also cause NCAM to be unable to accurately interpret the conclusions.

## 5. Conclusions

In summary, the highlight of our study is the discovery that low levels of circulating NCAM are closely related to the cognitive function of VaD. The level of circulating NCAM may be an indicator of the cognitive function of VaD patients. It is worthwhile for us to do clinical efficacy trials for intervention in NCAM in the future. The relationship between circulating NCAM levels and VaD cognitive function may provide a new treatment portal for VaD.

## Figures and Tables

**Table 1:** Clinical characteristics in controls and VaD patients.

	Controls (*n* = 83)	VaD (*n* = 98)	*p* value
Age (years)	67.4 ± 7.9	67.8 ± 8.1	0.738
Male gender (*n*, %)	56 (67.5)	70 (71.4)	0.564
Formal education (years)	10.6 ± 3.2	10.3 ± 3.4	0.544
Heart rate (bpm)	71.5 ± 12.7	70.8 ± 12.0	0.704
Supine systolic BP (mmHg)	138.2 ± 13.6	137.7 ± 13.8	0.807
Supine diastolic BP (mmHg)	81.4 ± 10.9	81.9 ± 11.1	0.761
FBG (mmol/L)	6.5 ± 0.4	6.6 ± 0.3	0.057
HDL cholesterol (mmol/L)	1.4 ± 0.5	1.3 ± 0.3	0.099
LDL cholesterol (mmol/L)	2.7 ± 0.4	2.8 ± 0.5	0.144
MoCA (points)	27.9 ± 1.4	23.0 ± 2.1	<0.001
NCAM (ng/mL)	21.7 ± 3.8	17.6 ± 4.2	<0.001

VaD: vascular dementia; BP: blood pressure; FBG: fasting blood glucose; HDL: high-density lipoprotein; LDL: low-density lipoprotein; MoCA: Montreal Cognitive Assessment; NCAM: neural cell adhesion molecule.

**Table 2:** Correlation between clinical characteristics and MoCA in patients with VaD.

	*r*	*p* value
Age (years)	-0.319	0.362
Male gender (*n*, %)	-0.283	0.445
Formal education (years)	0.472	0.236
Heart rate (bpm)	0.271	0.637
Supine systolic BP (mmHg)	0.118	0.302
Supine diastolic BP (mmHg)	0.184	0.143
FBG (mmol/L)	-0.362	0.593
HDL cholesterol (mmol/L)	0.299	0.108
LDL cholesterol (mmol/L)	-0.280	0.094
NCAM (ng/mL)	0.285	0.026

MoCA: Montreal Cognitive Assessment; VaD: vascular dementia; BP: blood pressure; FBG: fasting blood glucose; HDL: high-density lipoprotein; LDL: low-density lipoprotein; NCAM: neural cell adhesion molecule.

**Table 3:** Multivariable analyses of clinical characteristics and MoCA in patients with VaD.

	Regression coefficient	95% CI	*p* value
Age (years)	0.291	0.216-1.431	0.221
Male gender (*n*, %)	0.265	0.178-1.257	0.143
Formal education (years)	0.316	0.109-1.345	0.436
Heart rate (bpm)	0.117	0.036-1.134	0.380
Supine systolic BP (mmHg)	0.138	0.053-1.179	0.529
Supine diastolic BP (mmHg)	0.147	0.072-1.196	0.315
FBG (mmol/L)	0.330	0.128-1.310	0.137
HDL cholesterol (mmol/L)	0.246	0.167-1.183	0.412
LDL cholesterol (mmol/L)	0.209	0.097-1.202	0.318
NCAM (ng/mL)	0.223	0.101-0.768	0.034

MoCA: Montreal Cognitive Assessment; VaD: vascular dementia; BP: blood pressure; FBG: fasting blood glucose; HDL: high-density lipoprotein; LDL: low-density lipoprotein; NCAM: neural cell adhesion molecule.

## Data Availability

The data used to support the findings of this study are available from the corresponding author upon request.
